# Emotional and Clinical Aspects Observed in Women with Gestational Trophoblastic Disease: A Multidisciplinary Action

**DOI:** 10.1055/s-0042-1742681

**Published:** 2022-02-09

**Authors:** Ana Carolina Gomes França, Elza Maria Hartmann Uberti, Karine Paiva Muller, Rodrigo Bernardes Cardoso, Fabiana Giguer, Patricia El Beitune, Antonio Braga

**Affiliations:** 1 Universidade Federal de Ciências da Saúde de Porto Alegre, Porto Alegre, RS, Brazil; 2 Irmandade da Santa Casa da Misericórdia de Porto Alegre, Porto Alegre, RS, Brazil; 3 Universidade Federal do Rio de Janeiro, Rio de Janeiro, RJ, Brazil; 4 Universidade Federal Fluminense, Niterói, RJ, Brazil

**Keywords:** gestational trophoblastic disease, psychological aspects, support group, mental health, doença trofoblástica gestacional, aspectos psicológicos, grupos de apoio, saúde mental

## Abstract

**Objective** To evaluate the emotional and clinical aspects observed in women with gestational trophoblastic disease (GTD) followed-up in a reference center (RC) by a multidisciplinary team.

**Methods** Retrospective cohort study of the clinical records of 186 women with GTD and of the emotional aspects (EA) observed in these women by a team of psychologists and reported by the 389 support groups conducted from 2014 to 2018.

**Results** The women were young (mean age: 31.2 years), 47% had no living child, 60% had planned the pregnancy, and 50% participated in two or more SG. Most women (n = 137; 73.6%) reached spontaneous remission of molar gestation in a median time of 10 weeks and had a total follow-up time of seven months. In the group of 49 women (26.3%) who progressed to gestational trophoblastic neoplasia (GTN), time to remission after chemotherapy was 18 weeks, and total follow-up time was 36 months. EA included different levels of anxiety and depression, more evident in 9.1% of the women; these symptoms tended to occur more frequently in women older than 40 years ( *p* = 0.067), less educated ( *p* = 0.054), and whose disease progressed to GTN ( *p* = 0.018), as well as in those who had to undergo multi-agent chemotherapy ( *p* = 0.028) or hysterectomy ( *p* = 0.001) adjuvant to clinical treatment.

**Conclusion** This study found several EA in association with all types of GTD. It also highlights the importance of specialized care only found in a RC, essential to support the recovery of the mental health of these women.

## Resumo

**Objetivo** Avaliar aspectos emocionais e clínicos observados em mulheres com doença trofoblástica gestacional (DTG) acompanhadas em um centro de referência (CR), por equipe multiprofissional.

**Método** Estudo de coorte retrospectivo nos prontuários clínicos de 186 mulheres com DTG, e dos aspectos emocionais (AE) observados nessas mulheres pela equipe de psicólogas e registrados nos 389 grupos de apoio (GAs), ocorridos de 2014 a 2018.

**Resultados** As pacientes eram jovens (idade média 31,2 anos), 47% sem filhos vivos, 60% tinham desejado ou planejado esta gravidez e 50% delas participaram de dois ou mais GAs. A maioria (n = 137–73,6%) apresentou remissão espontânea da gestação molar com mediana de 10 semanas e um tempo total de seguimento de 7 meses. Quarenta e nove mulheres (26,3%) evoluíram para neoplasia trofoblástica gestacional (NTG); a mediana para atingir a remissão após tratamento com quimioterapia foi de 19 semanas e o tempo total de seguimento foi de 36 meses. Os AE incluíram variados graus de ansiedade e depressão, mais evidentes em 9,1% das nossas pacientes; tais AE tenderam a ocorrer mais em mulheres com idade acima de 40 anos ( *p* = 0,067), com menor escolaridade ( *p* = 0,054), com evolução para NTG ( *p* = 0,018), e nas que necessitaram de tratamento quimioterápico com regime de múltiplos agentes ( *p* = 0,028), ou de histerectomia complementar ao tratamento clínico ( *p* = 0,001).

**Conclusão** Este estudo mostrou presença de vários AE associados em todos os tipos de DTG. Destaca também a importância de um atendimento psicológico especializado, somente encontrado nos CR, que é essencial para ajudar na recuperação da saúde mental dessas mulheres.

## Introduction

The emotional aspects associated with normal pregnancies have been widely studied and described, particularly when associated with high-risk gestations that may pose special risks to maternal and fetal health. 1 2 Of all pregnancy complications in Brazil, about 1:200-400 are cases of gestational trophoblastic disease (GTD). GTD is a fertilization error of cytogenetic origin that potentially leads to an obstetric near miss and may progress to gestational trophoblastic neoplasia, which may be a cause of maternal death if not treated adequately. 3 4 5

These women face a great emotional impact when diagnosed with GTD. They experience the grief of a gestational loss, as well as the fear of being seriously ill and of losing their own life. 6 7 8 The difficulties that many women have in understanding a diagnosis of a molar gestation and its uncertain prognosis have social and psychological impacts that go beyond the diagnosis and treatment of GTD. 9 The emotional repercussions of this disease have been studied because of its particular circumstances and characteristics of GTD. 6 7 Their psychosocial impact should be understood to define directions for the improvement of the approaches adopted by multidisciplinary teams. 5 9

According to its natural history, GTD progresses to a cure most cases. However, its psychological stressors are real for both women and their partners. Both face unexpected changes of plans: the hopes and joys of pregnancy give way to the immediate need of adaptation to deal with a potentially serious disease. 10 11 12 Therefore, beyond medical care, GTD reference centers (GTD-RC), where women with GTD are followed up, organize support groups (SG) to provide psychosocial support to these women. 5 The objective of a SG is to contribute to health education and promote actions to improve women's mental health during the clinical follow-up of their disease. Some studies have demonstrated the efficacy of health education also in the case of GTD, as it clarifies questions and provides support to face the disease and its impacts. It also promotes adherence to treatments and anxiety control and provides support for their reproductive future. 5 9 13 14 15 16 17

Despite the importance of emotional aspects (EA) in its diagnosis, few studies have investigated the impacts of GTD, particularly the feelings associated with a pregnancy loss and a possible disease progression. Moreover, there are few studies about the potential benefits of psychotherapeutic interventions for these women. 3 4 5 6 7 8

This study evaluated EA associated with the diagnosis and follow-up of women with GTD, as well as with different clinical outcomes and treatment modalities. In some cases, the need of complementary individual psychological support and the use of psychoactive medication were required to restore the mental health of women with GTD.

## Methods

This is a retrospective cohort study to analyze data about EA collected from the records of 389 SG. Participants were 186 women with all types of GTD evaluated from 2014 to 2018 in the GTD-RC of the “Mario Totta” Maternity Ward of Irmandade Santa Casa de Misericórdia Hospital (ISCMPA), Porto Alegre, Brazil. Clinical and epidemiological data were reviewed in the medical records of the women seen in the GTD-RC, and qualitative data about EA were retrieved from the SG reports.

This study was approved by the ethics in research committee of the institution where it was conducted (CAAE 07388818.0.0000.5335 and CEP 3.209.698).

The SGs of the GTD-RC in the “Mário Totta” Maternity (MTM) of ISCMPA were first established in 1994 to provide psychosocial support in addition to medical care. 5 The SGs meet every seven to 14 days and are coordinated by the psychologist in the GTD-RC team. They also have the participation of a psychologist and a resident of the Gynecology and Obstetrics Department of ISCMPA or of the GTD-RC team. In the beginning of follow-up in the GTD-RC, all women and their families receive an explanation about the natural history of GTD and are invited and encouraged to participate in a SG. In the group, they have the opportunity to meet other women at different stages of GTD follow-up and have the chance to ask their questions and express their feelings and expectations about the disease and the future of their health.

At the time they are discharged from treatment in the GTD-RC, they are again invited to participate in a SG. At this moment, the idea is to have them share their experiences and life events and offer support and motivation to adhere to follow-up for women in the initial stages of post-molar follow-up.

When they return to the GTD-RC after a successful pregnancy, they are encouraged to participate in a SG once more, this time as representatives of a moment of hope of having a healthy pregnancy after the disease.

As the work in a SG is concluded, the psychologist in the group synthesizes the issues approached and reports on the EAs identified, which are later analyzed by the whole medical team responsible for the GTD-RC. For women that have more intense and frequent depressive symptoms or signs of anxiety, individual psychological care is offered, as well as a referral to a psychiatrist, whenever necessary. In the study period, the two psychologists who participated in this study estimated the frequency of each EA in women with spontaneous remission of GTD and in those whose disease progressed to GTN.

The data reviewed and collected from the medical records of the GTD-RC were age, age group, number of previous pregnancies, parity, number of living children, education, whether current pregnancy was planned, gestational age at diagnosis (weeks), GTD diagnostic method (imaging, clinical, histological), type of GTD (complete or partial hydatidiform mole, other GTDs), associated medical complications (such as anemia, blood transfusion, repeated curettage, laboratorial hyperthyroidism, pelvic infection and theca-lutein cysts), GTD progression (remission or GTN), time to remission (weeks), need of individual psychological care, depression or anxiety symptoms before GTN and during post-molar follow-up, total follow-up time (months) and discharge status.

GTD was diagnosed using the 2002 International Federation of Gynecology and Obstetrics (FIGO) criteria: hCG concentrations plateaued at values greater than or equal to 10%, hCG rise of more than 10% for three consecutive weeks, or histological diagnosis of choriocarcinoma. 18 19

Clinical staging of women that progressed to GTN used the 2002 FIGO-World Health Organization (WHO) criteria. Their risk score at the time of GTN diagnosis and beginning of the treatment indicated the most adequate type of management considering the risk of developing resistance to chemotherapy: single-agent chemotherapy when the risk score was lower than or equal to 6, and multi-agent chemotherapy if the score was greater than 7.

Quantitative variables were described as means and standard deviations or medians and interquartile ranges. Categorical variables were described as absolute and relative frequencies.

The Student t-test was used to compare means. The Mann-Whitney test was used for asymmetrical variables. Proportions were compared using the Pearson chi-squared or the Fisher exact test.

A multivariate Poisson regression model was performed to control for confounding factors. This model is appropriate to estimate prevalence ratios in studies when the outcome is dichotomous. Variables significant at *p* < 0.20 in bivariate analysis were included in the multivariate analysis, but only those significant at *p* < 0.10 were kept in the final model.

The level of significance was set at 5%, and the IBM SPSS Statistics for Windows, Version 21.0 (IBM Corp., Armonk, NY USA) was used for all statistical analyses.

## Results

Sociodemographic and clinical characteristics of the 186 women followed-up in the GTD-RC of the MTM/ISCMPA are shown in Table 1 . Mean age was 31.2 ± 9 years; about one third had only an elementary school education; almost half the women were nulliparous or had no living child; and pregnancy had been desired and planned in more than 60% of the cases. A possible molar pregnancy was investigated using ultrasound in 75% of the cases, and the definitive diagnosis was confirmed in 91.4% of these women. Except for transvaginal bleeding in 60% of the women, no other associated medical complications (such as anemia, blood transfusion, repeated curettage, laboratorial hyperthyroidism, pelvic infection, and theca-lutein cysts) were found in 75% of the women.

**Table 1:** Sociodemographic and clinical characteristics of the 186 women with GTD included in the study

Characteristics	n = 186	%
Age (in years) (mean ± SD, minimum - maximum)	31.2 ± 9	16–69
Education (N - %)		
Elementary (9 years)	52	28.0
Intermediate (12 years)	72	38.7
Higher (≥ 16 years)	62	33.3
Previous pregnancies (N - %)		
Zero	71	38.2
One	63	33.9
Two	52	28.0
Previous births (N - %)		
Zero	88	47.3
One	64	34.4
≥ Two	34	18.3
Living children (N - %)		
Zero	92	49.5
One	65	34.9
≥ Two	29	15.6
Planned or desired pregnancy (N - %)		
Yes	120	64.5
No	66	35.5
Gestational age (in weeks); mean ± SD, minimum - maximum	10.1 ± 3.8	4 - 29
Vaginal bleeding before diagnosis (N - %)		
Yes	111	59.7
No	75	40.3
GTD diagnostic method (N - %)		
Ultrasound	136	73.1
Histology only	50	26.8
Type of molar pregnancy (N - %)		
Complete hydatidiform mole	94	50.5
Partial hydatidiform mole	76	40.9
Other diagnoses	16	8.6
Associated clinical complications* (N - %)		
No	142	76.3
Yes	44	23.6

Abbreviation: GTD, gestational trophoblastic disease.

*associated clinical complications: anemia, blood transfusion, repeated curettage, laboratorial hyperthyroidism, pelvic infection and theca-lutein cysts.

GTD evolution is described in Table 2 , which shows that 50% of the women participated in two or more SGs. The disease progressed to GTN in 49 women (26.3%); almost 90% had GTN with a low risk of resistance to chemotherapy according to the 2002 FIGO/WHO criteria, and 83.6% of these cases were treated with only single-agent chemotherapy. As expected, both time to disease remission and time of follow-up were statistically greater in women with disease that progressed to GTN ( *p* < 0.001). Patient recovery was confirmed in 96.2% of the cases, and 79% were discharged after all post-molar follow-up was completed.

**Table 2:** Clinical evolution of the 186 women with GTD followed up in the study

Factors evaluated	N = 186 (%)	*p* -value
Participation in SG		< 0.001
One	94 (50.5)	
≥ Two	92 (49.4)	
Evolution		
Spontaneous remission	137 (73.6)	
GTN	49 (26.3)	
Time to remission (weeks)–median [P25–P75]***		
Spontaneous remission	10 (8-12)	
GTN	19 (16-26)	
FIGO/WHO risk score (n = 49)		
Low-risk	44 (89.8)	
High-risk	5 (10.2)	
Treatment (n = 49)		
Only chemotherapy	42 (85.7)	
Chemotherapy + surgery	7(14.2)	
Chemotherapy (n = 49)		
Single-agent	41 (83.6)	
Multi-agent	8 (16.3)	
Hysterectomy and indication (n = 49)		
No	42 (85.7)	
Yes	7 (14.2)	
Age, or childbearing completed	2 (28.5)	
Associated gynecological complications	4 (57.1)	
Recurrence	1 (14.2)	
Follow-up (months) - median (P25–P75)***		
Spontaneous remission	7 (6–9)	
GTN	36 (18–60)	
Discharge status		
Medical discharge after complete follow-up	147 (79.0)	
Loss to follow-up after remission (with normal hCG concentration)	32 (17.2)	
Loss to follow-up before remission (with elevated hCG concentration)	5 (2.7)	
Transferred	1 (0.5)	
Death	1 (0.5)	

Abbreviations: FIGO, The International Federation of Gynecology and Obstetrics; GTN, gestational trophoblastic neoplasia; hCG, human chorionic gonadotropin; SG, support group; WHO, World Health Organization.

***Mann-Whitney test ( *p* < 0.001).

Tables 3 and 4 show the association of EA related to depressive symptoms with women's clinical characteristics and GTD evolution variables. Grouped data revealed that 17 women (9.1%) had one or more depressive symptoms: six (3.2%) of them received individual psychological care; five (2.7%) had a history of depression; 16 (8.6%) had symptoms during follow-up; and 14 (7.5%) needed treatment with antidepressant medication. Data were grouped to obtain a larger sample size for the analysis of associations.

**Table 3:** Association between women's clinical characteristics and depressive symptoms

Variables	With depressive symptoms (n = 17)	Without depressive symptoms (n = 169)	*p* -value
Age (years)–mean ± SD	36.5 ± 10.9	30.6 ± 8.6	0.009*
Age range–n (%)			0.067**
≤ 19 years	0 (0.0)	11 (6.5)	
20–39 years	11 (64.7)	133 (78.7)	
≥ 40 years	6 (35.3) ^♪^	25 (14.8)	
Education–n (%)			0.054**
Elementary (9 years)	9 (52.9) ^♪'^	43 (25.4)	
Intermediate (12 years)	4 (23.5)	68 (40.2)	
College or higher (≥ 16 years)	4 (23.5)	58 (34.3)	
Planned pregnancy–n (%)	9 (52.9)	111 (65.7)	0.435**
Previous pregnancies–median (P25–P75)	1 (1–2.5)	1 (0–2)	0.012***
Number of births–median (P25–P75)	1 (0.5–2)	1 (0–1)	0.011***
Number of living children–median (P25–P75)	1 (0–2)	0 (0–1)	0.029***
Gestational age (weeks)–mean ± SD	9.9 ± 3.2	10.1 ± 3.9	0.827*
Type of molar pregnancy–n (%)			0.082**
Complete hydatidiform mole	11 (64.7)	83 (49.1)	
Partial hydatidiform mole	3 (17.6)	73 (43.2)	
Another diagnosis	3 (17.6)	13 (7.7)	
With associated medical complications–n (%)	4 (23,5)	40 (23.7)	1.000 ±

Abbreviations: GTN, gestational trophoblastic neoplasia; hCG, human chorionic gonadotropin; SD, standard deviation.

*Student *t* test; **Chi-squared test; ***Mann-Whitney test; ϮFisher exact test; ^#^ statistically significant adjusted residuals ( *p* < 0.05); ^♪^ results approaching significance.

**Table 4:** Association of GTD evolution with depressive symptoms

Variables	With depressive symptoms (n = 17)	Without depressive symptoms (n = 169)	*p* -value
GTD evolution–n (%)			0.018**
Spontaneous remission	8 (47.1)	128 (76.2)*	
GTN evolution	9 (52.9)*	40 (23.8)	
Time to remission (weeks) - median (P25–P75)	17 (12–27)	10.5 (8–15)	< 0.001***
GTN risk score - median (P25–P75)	6 (2–8.5)	2 (1–3)	0.008***
GTN Treatment - n (%) [N = 49]			0.224Ϯ
Only chemotherapy	7 (77.8)	37 (92.5)	
Chemotherapy and surgery	2 (22.2)	3 (7.5)	
Type of Chemotherapy–n (%) [N = 49]			0.028Ϯ
Single agent	5 (55.6)	36 (90.0) ^#^	
Multi-agent	4 (44.4) ^#^	4 (10.0)	
With adjuvant hysterectomy - n (%)	4 (23.5)	3 (1.8)	0.001Ϯ
Discharge conditions - n (%)			0.874**
Medical discharge after complete follow-up	15 (88.2)	132 (78.1)	
Loss to follow-up after remission, with normal hCG concentration	2 (11.8)	30 (17.8)	
Loss to follow-up before remission, with elevated hCG concentration	0 (0.0)	5 (3.0)	
Death/Transferred	0 (0.0)	2 (1.2)	
Follow-up (months)–median (P25–P75)	36 (9.5–66)	8 (6–12)	< 0.001***

Abbreviations: GTN, gestational trophoblastic neoplasia; hCG, human chorionic gonadotropin; SD, standard deviation.

*Student *t* test; **Chi-squared test; ***Mann-Whitney test; ϮFisher exact test; ^#^ statistically significant adjusted residuals ( *p* < 0.05); ^♪^ results approaching significance.

The multivariate analysis revealed that the tendency to depressive symptoms was about 2.5 times greater in women whose disease progressed to GTN ( *p* = 0.067) ( Table 5 ). Women that underwent hysterectomy had a three times greater probability of having depressive symptoms. For each child a woman had, this probability increased 59% (RR = 1.59).

**Table 5:** Multivariate Poisson regression to assess variables independently associated with depressive symptoms

Variables	Prevalence ratio (95%CI)	*p*
GTD evolution		
Spontaneous remission	1	
GTN	2.64 (0.94–7.44)	0.067
Hysterectomy	3.26 (1.11–9.58)	0.032
Number of living children	1.59 (1.09–2.33)	0.016

Abbreviation: GTN, gestational trophoblastic neoplasia.

Moreover, mean age of women whose disease progressed to GTN was significantly greater than that of those who had spontaneous remission (34.0 ± 10.9 vs 30.2 ± 7.9; p = 0.029). The women who had planned their pregnancies had a higher rate of return to medical care when pregnant again than those who had not (23.3% vs 9.1%; p = 0.027). Tables 6 and 7 show other important EA identified in the SGs, grouped according to GTD progression.

**Table 6:** Emotional aspects recorded in the SG for 137 women with spontaneous remission of GTD and no additional chemotherapy

Symptoms	%
Why me? —-—-—-—-—-—-—-—-—-—-—-—-—-—-—-—-—-—-—-—-—-----	90–95
Am I the only one with this disease? --------------------------------------------------------	80–95
Fear of the unknown --------------------------------------------------------------------------	85–90
Hard to explain the disease to others --------------------------------------------------------	10–45
Fear and guilt about fertility and next pregnancy ------------------------------------------	75–80
Helplessness about family–feeling alone and sad when facing the disease -----------	50–60
Inability to feel hurt about pregnancy loss --------------------------------------------------	20–30
Powerlessness in face of future motherhood (especially women without children)-------------------------------	40–95
Desire to have sterilization out of fear of next pregnancy -------------------------------	0–50
Social shame: ashamed of facing social groups (workmates, friends, neighbors) -------------------------------	20–90
Emotional difficulties during a new pregnancy --------------------------------------------	50–80
Awareness of risks of a new pregnancy -----------------------------------------------------	90–95
Ambiguous feelings about a new pregnancy: fear and desire ----------------------------	5–20
Sexual dysfunction -----------------------------------------------------------------------------	10–30
Ambiguous feelings about the attending team ---------------------------------------------	2–5
Feeling that care received out of the RC was not supportive ----------------------------	70–95
Feeling angry and ashamed of feelings when seeing other pregnant women ---------	75–95

**Table 7:** Emotional aspects observed in the SG in 49 women whose disease progressed to GTN and who needed chemotherapy

Symptoms	%
Guilt for being ill --------------------------------------------------------------------------------	10–40
Fantasizing about cancer and death -----------------------------------------------------------	60–95
Fantasizing about crying or suffering, believing they will make disease get worse ----	20–90
Low self-esteem because of body image (hair loss) ----------------------------------------	70–95
Helplessness about surgery (hysterectomy) --------------------------------------------------	10–95
Ambiguous feelings about being ill and weak inside while looking strong outside ----	30–80
Fear about living with a “damaged” uterus ---------------------------------------------------	5–10
Feeling of inferiority and of being unable to bear children --------------------------------	50–70
Difficulty in interacting socially after chemotherapy ---------------------------------------	40–80
Use of disease for secondary gain and guilt relief -------------------------------------------	40–65
Fear and guilt about fertility and next pregnancy --------------------------------------------	80–95
Fear and denial of death -------------------------------------------------------------------------	10–30

A high level of anxiety was a constant finding before admission to the RC. After admission, some actions, such as acquisition of an accurate knowledge of the natural history of GTD, personal meetings with other women experiencing the same disease and participation in specific social media platforms, such as the Facebook group in the profile of the Brazilian Association of Gestational Trophoblastic Disease, helped reduce anxiety.

## Discussion

Disease progression to GTN, hysterectomy adjuvant to GTN treatment and living child were the main stressors independently associated with depressive symptoms among the women with GTD followed up in this study.

Other studies have investigated the emotional impact of disease on women with GTD using standardized questionnaires, such as the Quality of Life, Satisfaction with Life and the WHO Quality of Life scales, as well as the evaluation of depressive symptoms. However, a more detailed analysis of these symptoms and of the emotional repercussions of the disease requires an accurate evaluation using qualitative data about grief, death, anger, and delay of childbearing plans. 19

Anxiety symptoms may also have been reported in the form of ignorance and/or fear of the disease, sexual dysfunction, low level of maternal self-efficacy, guilt for being sick and low self-esteem, 20 as showed in Tables 6 and 7 .

Several studies have reported on the relevance of the impact of communications, treatment and emotional support received from a multidisciplinary team. 6 17 18 20 The perceptions of women with GTD have also been evaluated using questionnaires, such as the Health-Related Quality Of Life and the Patient Reported Outcome Measures (PROM), which cover several domains, such as physical effects, emotional symptoms (anxiety, depression) and social relationships. Psychological interventions, participation in a SG and participation of the family, when combined with continuous education about their disease, play an important role in the context of the treatment by a multidisciplinary team. 3 7

Ferreira et al. 11 found that the lowest scores of quality of life were associated with emotional effects, whereas physical effects due to multi-agent chemotherapy did not affect it significantly. 21 Our results showed that hysterectomy, a surgical adjuvant treatment and the most important physical intervention in our study, was significantly associated with depressive symptoms. Women whose disease progressed to GTN had more symptoms of anxiety and stress, but chemotherapy had a curative effect on patient survival and did not limit their future childbearing plans or made their reproductive future worse. 22 23

Many studies found significant numbers of depressive symptoms in women with GTN. 6 8 24 Feelings after a molar gestation, similar to the grief felt in case of miscarriages, include depressive symptoms and reactions. 5 Several anxiety symptoms are associated with the weekly or regular measurement of the tumor marker (hCG). 8 Problems associated with relationships, marriage and reproductive future also seem to point to a greater level of sexual dysfunction in women with GTD. 24 25 No association with infertility has been found, and most women have a positive reproductive performance, confirmed by subsequent term pregnancies without complications. 8 26

Study comparisons are limited by the difficulty in comparing heterogeneous qualitative details, although some studies have used standardized objective questionnaires. Regardless of severity of psychological effects, the emotional support of a multidisciplinary team is a unanimously effective tool to promote well being and improve women's quality of life. 27 28 The participation in SGs also strengthens adherence to follow-up and return for the visits at the recommended regular intervals. Multidisciplinary teams have an important role in detecting depressive symptoms and managing the psychological repercussions of treatment on both the women and their families during follow-up. 7

In the future, studies about the psychosocial aspects of GTD will probably use PROM questionnaires. This instrument may be used to evaluate patient health at different time points of their follow-up, differently from other questionnaires that evaluate only disease and outcome. 22 29 30

One of the limitations of this study is the fact that, as a retrospective study, it is difficult to characterize EA accurately, as SG records have been made along many years. Moreover, the longer time to remission and follow-up time of women whose disease progressed to GTN are confounding variables that may affect the prevalence of depressive symptoms.

Short- and long-term problems are associated with the quality of life and the psychological, social, and sexual consequences of a diagnosis of GTD, particularly when the disease progresses to GTN. Moreover, women suffering because of their experience in a hospital context, the fragility of their physical health, the constant return visits to a place marked by disease, the chances of deterioration or a prognostic change should all be taken into consideration in this analysis.

## Conclusion

The main objectives of GTD-RCs, which provide multidisciplinary care to women with all types of GTD, are the recovery of physical and mental health, the preservation of fertility and the improvement of quality of life. Therefore, women with GTD should be referred to follow-up in one of the 41 GTD-RCs in Brazil at an early stage. Follow-up in a specialized RC contributes to reducing morbidity and mortality. Moreover, it promotes positive follow-up experiences, provides understanding and mitigation of psychological stressors of treatment, and ensures that treatment approaches are comprehensive and focused on the women and their families.

## Contributors

All authors were involved in the design and interpretation of the analyses, contributed to the writing of the manuscript, and read and approved the final manuscript.
